# The neurophysiology of central and peripheral fatigue during sub-maximal lower limb isometric contractions

**DOI:** 10.3389/fnhum.2013.00135

**Published:** 2013-04-15

**Authors:** Marika Berchicci, Federica Menotti, Andrea Macaluso, Francesco Di Russo

**Affiliations:** ^1^Department of Human Movement, Social and Health Sciences, University of Rome “Foro Italico”Rome, Italy; ^2^Neuropsychological Unit, Santa Lucia Foundation IRCCSRome, Italy

**Keywords:** movement-related cortical potentials (MRCPs), rating of perceived efforts (RPE), isometric contraction, maximal voluntary contractions (MVC), maximal twitch (MT)

## Abstract

Fatigue has been defined as an exercise-induced decline in force generation capacity because of changes at both the peripheral and central levels. Movement is preceded and accompanied by brain activities related to the preparation and execution of movement (movement related cortical potentials, MRCP), which have been correlated with the perception of effort (RPE). We combined force measurements, surface electromyography (sEMG), peripheral electrical stimulation (maximal twitch, MT) and MRCP analysis to further our understanding of the neural correlates of peripheral and central changes during a fatiguing task involving the lower limbs. Eighteen healthy volunteers performed 4 blocks of isometric knee extensions at 40% of the maximal voluntary contraction (MVC) for a total of 240 2-s contractions. At the baseline and after each block, we measured RPE, MT and MVC. We simultaneously recorded the force of the knee extensor muscles, root mean square (RMS) of the sEMG of the vastus lateralis muscle, and electroencephalography (EEG) from 64 channels. The MRCPs were extracted from the EEG recordings and averaged in the early (Block 1–2) and late (Block 3–4) blocks. Two cohorts were obtained by cluster analysis based on the RPE (i.e., perception of effort) and MT (i.e., peripheral fatigue). We observed a significant decline in both the MVC (−13%) and RMS (−25%) of the sEMG signal over the course of the task; thus, muscle fatigue had occurred in all of the participants regardless of the cohort. The MRCP amplitude was larger in the fatigued than the non-fatigued MT cohort in the supplementary and premotor areas, whereas the MRCP amplitude was larger in the fatigued than the non-fatigued RPE cohort in the aforementioned areas, and also in the primary motor and prefrontal cortices (PFC). The increase in the positive activity of the PFC, along with the perception of effort, represents a novel result, suggesting that it is modulated more by the perception of effort than peripheral fatigue.

## Introduction

Fatigue is a multidimensional concept combining physiological and psychological aspects. In physiology, fatigue is usually defined as a time-related loss of voluntary force-producing capacity during physical exercise (Gandevia, 2001). The maximal voluntary contraction (MVC) measured at different stages of the fatiguing task shows a gradual decline in maximal force-generating capacity during submaximal exercise, even though the target force can still be maintained (Zijdewind et al., 1998; Søgaard et al., 2006). The degree of fatigue in the muscle itself, which we refer to as peripheral fatigue, may be estimated by using electrical stimulation (maximal twitch, MT) at rest (see Enoka and Duchateau, 2008 for a review). Fatigue has also been studied by measuring neural activation of the peripheral muscle from surface EMG, which reflects the overall number, firing rate, and synchronization of the active motor units (Menotti et al., 2012). Fatigue often begins gradually soon after the beginning of the contractions, even though the individual is able to continue the task (Søgaard et al., 2006; Barry and Enoka, 2007); thus, fatigue, task failure, and exhaustion should be distinguished. The decline in force-producing capacity may originate from various levels of the neural axis, including the muscle metabolism and membrane, the neuromuscular junction, or the motor cortex (Allen, 2004; Zwarts et al., 2008).

Fatigue includes decline in muscle force, whether the task can be sustained, and encompasses sensations that relate to tasks being more difficult or taking more effort than expected (Taylor and Gandevia, 2008). Borg (1982) has developed a psychophysical scale with ratio properties to quantify the perception of effort during physical activities, which allows direct comparisons with physical and physiological measurements. This scale has been developed for differential use and is able to cover a reasonable range of intensities (e.g., lifting weights, sub-maximal contraction, walking, or running). The rating of the perceived effort (RPE) scale ranges from 0 (no effort at all) to 10 (maximal effort), and to make the numbers usable and clear to most people, the numbers are anchored by standard verbal expressions from *nothing at all*, *moderate*, *strong*, *to very very heavy* (Borg, 1982). Most of the studies that are interested in understanding the central and peripheral mechanisms of neuromuscular fatigue during isometric muscle contraction (Sjøgaard et al., 1991; Allman and Rice, 2003; Dirnberger et al., 2004; Slobounov et al., 2004; Hummel et al., 2005; Lambert et al., 2005; Søgaard et al., 2006; Heuser and Pincivero, 2010; Falvo et al., 2011; Hotta and Ito, 2011; Pincivero, 2011; De Morree et al., 2012) have assessed the perception of effort by using the Borg category ratio scale (CR-10) (Borg, 1982, 1990, 1998), also known as the RPE scale.

The perception of effort refers to all of the subjective sensations accompanied during an exercise performance; thus, the increased perception of effort might be associated with different patterns of fatigue depending on the exercise. However, these sensations play a key role in maintaining physical integrity and represent psychological entities that introduce changes in behavior through a central strategy (see Ament and Verkerke, 2009 for a review).

One way to study the brain correlates of fatigue is through the use of the motor-related cortical potential (MRCP), which is a class of event-related potentials (ERPs) locked on the initiation of movement. These electro-cortical activities are particularly useful, as the conscious intention to move, sense of agency and, therefore, conscious sensation of effort are preceded by brain activities in the prefrontal cortex, supplementary, premotor and primary motor areas, and somatosensory and posterior parietal cortices (Desmurget and Sirigu, 2009; Haggard, 2009; Preston and Wegner, 2009; Bozzacchi et al., 2012a,b). The magnitude and timing of activity in these brain areas are reflected by the amplitude and latency of the MRCPs, which can be considered a direct neurophysiological measure of central motor command. Freude and Ullsperger (1987) introduced the use of the MRCP, elsewhere referred to as Bereitschaftspotential (BP) or readiness potential (RP), as a tool to determine changes at the motor cortex level during fatiguing contractions. The earliest component of the MRCP, the BP, is characterized by a gentle rise in negativity starting more than 1 s before movement initiation. The authors found that the BP amplitude increases during repetitive contractions at both low and high force levels, which has been explained as a way to compensate for peripheral fatigue. Several authors replicated these findings (Johnston et al., 2001; Dirnberger et al., 2004; Siemionow et al., 2004; Liu et al., 2005; Schillings et al., 2006), while fewer and more dated studies observed an attenuation of the MRCP (Kristeva, 1977; Popivanov et al., 1995), which was interpreted as a decreased intentional involvement and habituation processes. Of more interest, there are two studies that confirmed the original observation of Freude and Ullsperger and also found positive relations between the MRCP amplitude and amount of perceived effort (Slobounov et al., 2004; De Morree et al., 2012), suggesting a relevant influence of cognitive processing on the development of fatigue. However, in all of these studies, the fatigue-related effect on MRCP was observed in the frontal areas over the supplementary, premotor and primary motor areas. No activity has been observed in the prefrontal and posterior parietal areas. The explanation for the lack of activity in these cognitive areas may be twofold: (1) tasks such as force handgrip contractions, weight lifting with the elbow flexors, or isometric finger contractions, which require motor control of the smaller muscle groups and less executive/inhibitory processing during the planning of the action when compared to the contraction of the lower limbs; (2) the experimental approaches did not distinguish between the cortical mechanisms underpinning both the subjective and objective fatigue.

Although many concepts are understood regarding the physiology of fatigue of the upper limbs, studies on the lower limb muscles are lacking. We combined force measurements, surface electromyography (sEMG), peripheral electrical stimulation, and MRCP analysis to further our understanding of the neural correlates of peripheral and central changes during fatiguing tasks involving the large muscle mass of the quadriceps. The aim of this study was to replicate previous findings concerning the correlation between the MRCP and RPE during a fatiguing task and extend those results by including the analysis of each MRCP component preceding and accompanying the submaximal isometric contractions. Furthermore, with the aim to observe activity in the prefrontal and posterior parietal areas, we designed a fatiguing task that was also cognitively demanding because the participant had to produce time-restricted submaximal isometric contractions with the leg on the basis of visual feedback. We hypothesized that the participants with both high perception of effort and a high loss of MT after the fatiguing contractions would show an increase in the MRCP components over the frontal areas, while only those with high RPE would show modulation in the prefrontal and/or posterior parietal areas. To the best of our knowledge, this experimental approach has not previously been used to investigate the effects of muscle fatigue and perception of effort on MRCP components.

## Materials and methods

### Participants

Twenty-seven healthy volunteers were studied. However, because of the high number of artifacts in the electroencephalography (EEG), the data from 9 participants were discarded; thus, the data refer to a total of 18 participants (10 men, mean age 30.3 ± 9.9 years, mean body mass 66.7 ± 8.9 kg) without a history of psychiatric/neurological disease and musculoskeletal injury. All participants had normal and/or corrected vision. Each participant provided written informed consent prior to testing. The protocol was approved by the local ethics committee and was performed in accordance with the Declaration of Helsinki.

### Force and EMG recording and analysis

The torque of the knee extensor muscles of the dominant limb was measured using a force transducer (Model 9203; Kistler, Winterthur, Switzerland). The force signal was amplified (×1000) using a charge amplifier (Type 5011; Kistler) displayed in front of each participant on an oscilloscope (TDS 220; Tectronix, Beaverton, Oregon). The charge amplifier was connected to both the EMG amplifier and EEG recording systems for signal synchronization and then stored on a PC.

The signals from sEMG were recorded using adhesive linear arrays of four electrodes (silver bars 5 mm long, 1 mm thick, and 10 mm apart; LISiN, Torino, Italy) from the vastus lateralis muscle. This configuration allowed for the detection of 3 sEMG signals in a single-differential mode from each electrode array. After a light skin abrasion that was cleaned with alcohol, the arrays were positioned on the muscle belly halfway between the innervation zone and distal tendon along the estimated orientation of the muscle fibers. A conductive gel was inserted with a syringe into the grooves of the adhesive electrode arrays for each electrode to ensure proper electrode–skin contact. A ground electrode was placed on the patella bone of the same limb. Before final electrode positioning, translation of the action potentials was visually verified. The sEMG signal was amplified (×1 K) and filtered (band-pass 10–500 HZ) using a multichannel EMG amplifier (EMG USB2, OT Biolelettronica, Turin, Italy). Both sEMG and force signals were sampled at 2048 HZ by the EMG amplifier and stored on a PC for further analysis.

Electrical simulation was used to measure the MT of the studied muscle in different stages of the fatiguing protocol. The stimulation was carried out by placing a cathode electrode (5 × 10 cm) on the proximal third of the quadriceps femoris muscle and an anode electrode distally on the muscle-tendon junction. Single impulses lasting 200 μs with a monophasic rectangular wave and constant envelope were delivered with a high voltage stimulator (Digitimer DS7A/AH, Welwyn Garden City, UK). MT assessment consisted of delivering a sequence of single impulses to the quadriceps muscle in increments of 10 mA amplitude and separated by 30 s of rest until the maximal-mechanical response was obtained from each participant (i.e., the range of stimulus intensities ranged between 150 and 210 mA).

All of the data were analyzed off-line using OT Biolab software (OT Biolelettronica, Turin, Italy). The torque was calculated as the product of the force recorded by the transducer and the distance between the axis of rotation of the joint and the point where the force was applied. The MVC torque was chosen as the mean value of a 1 s window around the peak torque, and the twitch torque was chosen as the peak torque value. The sEMG signals obtained from a single array during MVC were computed on 2 double differential signals over epochs of 250 ms. To quantify the EMG amplitude, expressed as the root mean square (RMS), a computer-aided analysis was performed over the 1 s epoch corresponding to the MVC. This procedure has been described in detail elsewhere (Macaluso et al., 2000).

### EEG recording and analysis

Continuous EEG was recorded using the BrainVision™ system (BrainProducts GmbH., Munich, Germany) with 64 active sensors (ActiCap™ BrainProducts GmbH., Munich, Germany) and mounted according to the 10-10 International System, which was initially referenced to the left mastoid. The EEG was digitized at 250 Hz, amplified (bandpass of 0.01–80 Hz including a 50 Hz notch filter) and stored for off-line averaging. Horizontal eye movements (electro-oculogram, EOG) were monitored with bipolar recordings from electrodes at the left and right outer canthi. The blinks and vertical eye movements were recorded with an electrode below the left eye, which was referenced to site Fp1. The participants were required to concentrate on the task performance and minimize distractions as much as possible. They were asked to maintain a stable body position and avoid eye blinks, teeth clenching, and upper limb and head movements during leg contractions, whereas eye blinks and body adjustments were allowed during the inter-trial periods. Possible sources of distraction and noise were minimized.

Offline analysis was performed utilizing the BrainVision™ Analyzer 2.0.1 software (Brain Products GmbH., Munich, Germany). Raw EEG data were re-referenced to average mastoids and visually inspected to identify and discard epochs contaminated with artifacts prior to the signal averaging. The trials with artifacts (e.g., blinks or gross movements) were automatically excluded from the averaging, whereas eye movement artifacts were corrected by the Gratton et al. algorithm (1983). For each trial, the onset of contraction, which was defined as the time at which the force curve exceeded the baseline force by approximately 15 N (Suetta et al., 2004), was sent to the EEG recording system.

The MRCPs were separately segmented and averaged into non-overlapping 2500 ms-epochs that were measured 2000 ms before and 500 ms after the onset of contraction. For each data set, the grand average was calculated. The baseline was derived from the mean amplitude over the initial 300 ms of the averaged epochs. To further reduce high-frequency noise, the time grand-averaged MRCPs were low-pass filtered at 15 Hz. The MRCP onset latency was calculated as the first deflection larger than twice the absolute value of the baseline mean. According to previously published reports (e.g., Shibasaki and Hallett, 2006), the three components of the pre-contraction slow waves were calculated in the following manner. The BP was derived by calculating the mean amplitude from 1000 ms to 500 ms before the onset of contraction. The *negative slope*, or NS', was calculated as the mean amplitude from 400 ms to 100 ms before the onset of contraction. The *motor potential*, or MP, was calculated as the mean amplitude between 100 ms before and the initiation of the isometric contraction. The aforementioned parameters were measured on the peak electrodes (e.g., channels with maximal amplitude) and selected for statistical analysis because the parameters are known to reflect the distinct stages of preparation, planning, and initiation of the actions in the premotor and motor brain areas (e.g., Shibasaki and Hallett, 2006; Berchicci et al., 2012; Bozzacchi et al., 2012a,b). The selection of the electrodes was also based on the scalp topography, which allowed us to identify the electrodes where the signal was maximal.

To visualize the voltage topography of the MRCP components, spline interpolated 3-D maps were constructed using the BESA 2000 software (MEGIS Software GmbH, Gräfelfing, Germany).

### Procedure

**Figure 1:**
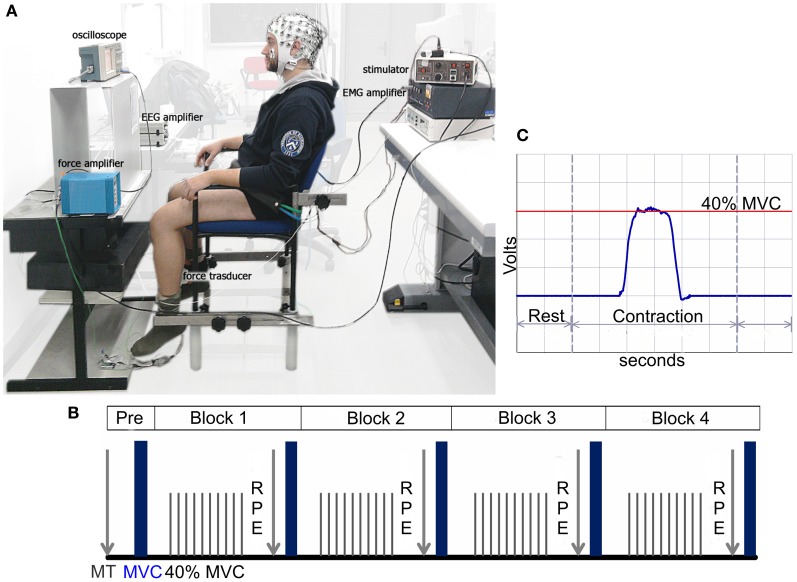
**(A)** Experimental setting with the participant positioned during simultaneous EEG, EMG, and dynamometric recordings. **(B)** Schematic representation of the experimental protocol. The “Pre” phase refers to the maximal twitch (MT) and maximal voluntary contraction (MVC) determined before each experimental session as a baseline. Each block includes 60 self-paced isometric contractions at 40% of the MVC followed by the rating of perceived effort (RPE), MT, and MVC. **(C)** Real-time visual force feedback regarding the subject's current force level produced by the leg displayed on the oscilloscope screen.

The participants were seated on a custom-made chair and stabilized with a waist belt. The chair was positioned upright, and both the hip and knee angles were set at 90°. The frontal side of the leg just above the ankle was in contact with a support linked to a fixed force transducer. This position allowed the participants to exert isometric contractions of the knee extensor muscles in the sagittal plane (see Figure 1A). All of the participants were given a practice session to become familiar with the procedures and establish a stable visuo-motor strategy consistent with the task requirements to minimize eye movement, especially prior to contraction. During the practice session, the EEG and fatiguing setup were explained to the participants in detail by the experimenters. The participants were then provided with standardized instructions about the self-report perception of effort assessed with a modified Borg category-ratio (CR-10) scale or RPE scale (Borg, 1982, 1990, 1998), which we refer to as RPE.

Once the experimental setup was completed, the MT and MVC were determined before each experimental session (e.g., baseline or Pre in Figure 1B). A sequence of single stimuli was delivered to the quadriceps muscle that gradually increased the amplitude of each stimulus. A resting period was given between the stimulations for adequate recovery. The stimulation with the highest peak torque was chosen as the MT. To assess the MVC, the participants were asked to isometrically extend the knee joint by pushing against the load cell as hard as possible for 2–3 s before relaxing. The participant performed three attempts with rest intervals of 5 min, and the strongest MVC was used. Further attempts were requested if the MVC of the last trial exceeded the previous one by at least 10%.

The fatiguing task consisted of 240 intermittent isometric leg contractions at 40% of the participant's MVC. The contractions were performed during four blocks of 60 contractions each, with a brief pause (<2 min) separating the tests where the RPE, MVC (one attempt) and MT (one pulse) were measured (Figure 1B).

Each participant was presented with a horizontal target line on the oscilloscope screen representing 40% of his/her MVC. The screen was placed at eye-level approximately 60 cm in front of the participant. To maintain the appropriate contraction intensity, real-time visual force feedback regarding the subjects' current force level (cursor of the force trace) produced by the leg was provided to the subject on the screen (Figure 1C). The participant was instructed to perform an isometric sub-maximal contraction reaching the target force and matching their force trace with the target line as smoothly and accurately as possible for approximately 2 s. The contractions were self-paced, and the subject was instructed to maintain a time interval between the trials that was approximately 10 s.

### Statistical analysis

Because the main purpose of the present study was to better understand the neural mechanisms underpinning the changes along the sensorimotor system and muscle tissue during a fatiguing task, the participants were divided into different cohorts based on the following criteria: (1) the RPE, which reflects the perception of effort, and (2) the MT, which is related to muscle fatigue. Cluster analysis was performed separately for RPE and MT (both recorded in all of the participants after each block of contractions) to identify any natural grouping that may exist in the involved sample of individuals. A two-step cluster analysis was used, and the distance measures based on pattern similarity were chosen in forming the clusters. After cluster analysis, the participants were divided into the non-fatigued (i.e., NF) and fatigued (i.e., F) cohort based on the RPE and MT, thus obtaining the following cohorts: NF-RPE and F-RPE cohort, NF-MT and F-MT cohort. Therefore, all of the further analyses were separately performed for the NF- and F-RPE cohort and the NF- and F-MT cohort.

The 240 intermittent isometric leg contractions performed at 40% of the MVC were divided into two blocks of trials, one early (Block 1 and 2) and one late (Block 3 and 4) to obtain a sufficient number of trials and an adequate signal-to-noise ratio in the MRCP.

Quantitative differences in the force and sEMG data (MVC and RMS) were investigated using a 2 × 4 ANOVA, with Group (NF vs. F) as the between-subjects factor and Block (1 vs. 2 vs. 3 vs. 4) as the within-subjects factor.

The MRCP onset latency data were statistically analyzed using the Fp1 electrode, which showed the earliest activity among all of the groups and conditions. The onset latency was analyzed using a 2 × 2 ANOVA using Group (NF vs. F) as the between-subjects factor and Block (early vs. late) as the within-subjects factor.

The following electrodes were selected for the statistical analysis of the MRCP amplitude based on the peak amplitude of each component and the previous studies mentioned in the introduction section: for the BP component FC1 (roughly overlaying contralateral supplementary motor area), for the NS' was selected Cz (roughly overlaying premotor areas), and for the MP was selected C1 (over the contralateral primary motor cortex); for the prefrontal positivity (pP) were selected Fp1 and Fp2 over the prefrontal cortex. For each of the components and the relevant electrode, a 2 × 2 ANOVA was performed using Group (NF vs. F) and Block (early vs. late) as the factors. ANOVAs were performed for the groups defined as RPE and MT. *Post-hoc* comparisons were conducted using Tukey's HSD test. The overall alpha level was fixed at 0.05 after the Geisser-Greenhouse correction.

The correlation coefficients (Pearson's *r* coefficients) were computed in the whole sample (*N* = 18) between the RPE scores at block 4 and MRCP components' amplitude (i.e., BP, NS', MP, and pP) at the peak electrodes during the late blocks. The significance was set at 0.05 (two-tailed) for all analyses.

## Results

### Subject clustering

Cluster analysis of the MT recorded after the 240 sub-maximal contractions (i.e., the MT of block 4) led to the definition of two distinct clusters: one cluster (*N* = 8; mean age 33.8 ± 11.2 years) was characterized by a low MT (F), while the other cluster (*N* = 10; mean age 27.4 ± 8.4 years) was characterized by a high MT (NF) expressed as a percentage of the baseline. Individuals in the F-MT cluster showed significantly lower MT scores (66.1 ± 12.9) than the individuals in the NF-MT cluster (89.9 ± 3.3; *p* < 0.05).

Cluster analysis of the RPE after 240 contractions (i.e., the RPE of block 4) led to the definition of two distinct clusters: one cluster (*N* = 9; mean age 28.3 ± 8.2 years) was characterized by a high RPE (F), while the other cluster (*N* = 9; mean age 32.2 ± 11.7 years) was characterized by a low RPE (NF). Individuals in the F-RPE cluster showed significantly higher RPE scores (5.8 ± 1.1) than the individuals in the NF-RPE cluster (3.0 ± 1.3; *p* < 0.05).

Further analysis showed that the RPE at the beginning of the fatiguing protocol (after Block 1) was not significantly different between the F-RPE and NF-RPE cohorts (3.0 vs. 2.0, respectively, *p* = 0.089). In addition, the ΔRPE, calculated as the difference between the RPE of block 4 and block 1 was greater in the F-RPE than the NF-RPE cohort (2.5 vs. 1.0, *p* = 0.01).

### Force and EMG data

**Figure 2:**
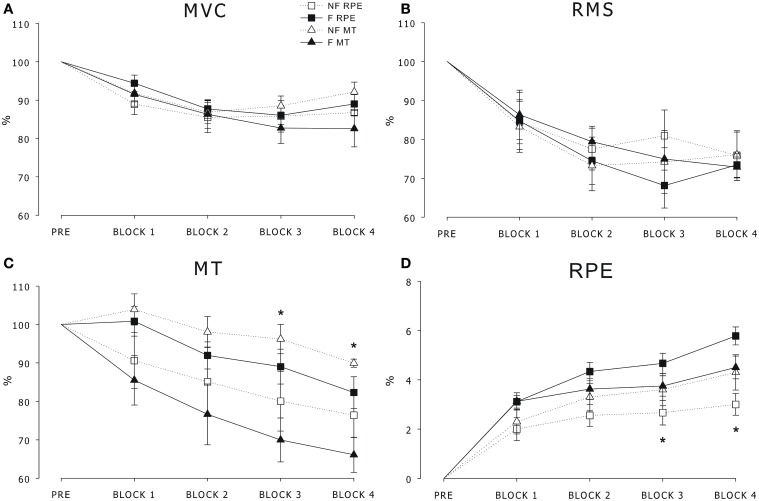
**(A)** Maximal voluntary contraction (MVC) force, **(B)** root mean square (RMS) of the EMG signal, **(C)** maximal twitch (MT) and **(D)** rating of perceived effort (RPE) over time in the F-MT and NF-MT cohorts (triangles) and F-RPE and NF-RPE cohorts (squares). Data (Mean ± SE) are reported as the percentage of the PRE values. ^*^significant difference between the fatigued (F) and non-fatigued (NF) cohorts (*p* < 0.05).

All of the participants were able to perform the task throughout the protocol, and at the sub-maximal contraction steady-state, the force was maintained in all of the participants with an average of 38.29% of the pre MVC and a duration of 1.37 s. Statistical analysis showed a main effect of Block in both the MVC and RMS [*F*_(3, 48)_ > 3.23, *p*s < 0.05] as reported in Figures 2A,B. The Group effect and interaction were not significant, thus suggesting a similar effect of fatigue across the sub-maximal contractions in all of the participants regardless of clustering. Statistical analysis showed a main effect of Block in the MT cohorts [*F*_(3, 48)_ > 15.21, *p*s < 0.05] as shown in Figure 2C. The *post-hoc* analysis revealed that the MT was higher in the NF-MT with respect to the F-MT in block 3 and block 4. Similarly, the statistical analysis showed a main effect of Block in the RPE cohorts [*F*_(3, 48)_ > 15.21, *p*s < 0.05], as shown in Figure 2D. The *post-hoc* analysis revealed that the RPE was lower in the NF-RPE with respect to the F-RPE in block 3 and block 4.

### MRCP data

#### Waveforms

**Figure 3:**
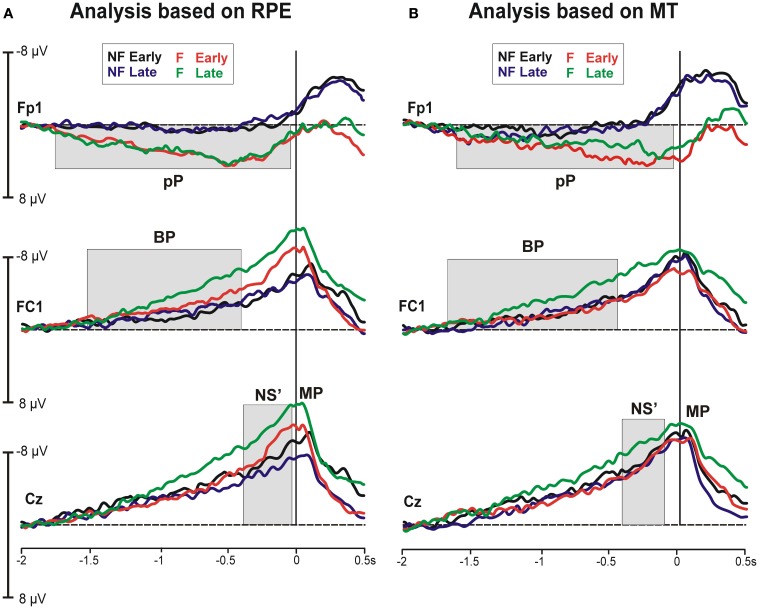
**Grand average MRCP waveforms at the prefrontal (Fp1), precentral (FC1), and central (Cz) electrodes based on the rating of perceived effort (A) and the maximal twitch (B) during the early and late blocks of isometric contractions**.

Figure 3 shows the representative grand averaged MRCP waveforms at the prefrontal (Fp1), precentral (FC1) and central (Cz) sites, where the MRCP components were maximal. Waveforms after the early and late blocks were superimposed for the participants who reported a higher vs. lower increase in effort (analysis based on the RPE, Figure 3A) and participants with a higher vs. lower decrease in MT (analysis based on the MT, Figure 3B). A common pattern was visible in the four cohorts of participants. This pattern indicated that the typical MRCP component was the early BP, which was characterized by a gradual increase in negativity starting at approximately 1500 ms before the contraction and was most prominent over FC1. This negativity increased approximately 500 ms before contraction onset (NS'), peaking around the time of initiation of the isometric contraction (MP). Furthermore, a consistent slow rising and very early positivity was also present at the prefrontal sites, especially in the F-RPE cohort. This activity initiated at approximately −1700 ms, peaked at approximately −500 ms and returned to baseline concomitant with the contraction. Statistical analyses on this component were performed on 500 ms time windows from −700 to −200 ms. Hereafter, this activity will be called *prefrontal positivity* (i.e., pP).

#### Analysis based on RPE

Statistical analysis of the MRCP onset latency (on Fp1) showed a significant Group effect [*F*_(1, 16)_ = 14.41, *p* = 0.001] occurring much earlier in the fatigued (−1668 ± 82 ms) than non-fatigued (−1104 ± 185 ms) cohort. The Block effect and interaction were not significant.

The BP amplitude (on FC1) revealed main effects of both the Group [*F*_(1, 16)_ = 5.29, *p* = 0.035] and Block [*F*_(1, 16)_ = 6.51, *p* = 0.021], indicating that the BP was larger in the fatigued cohort and late blocks. The interaction was not significant. Analysis of the NS' amplitude (on Cz) revealed significant main effects of Block [*F*_(1, 16)_ = 6.03, *p* = 0.025], with larger negative amplitude at late (−11.01 μV ± 1.21) than early (−8.71 μV ± 1.06) blocks. The Block effect and interaction were not significant. Analysis of the MP (on C1) revealed significant main effects of Group [*F*_(1, 16)_ = 6.58, *p* = 0.020], with a larger negativity for the fatigued than non-fatigued cohort. The Block effect and interaction were not significant. The pP amplitude showed a main effect of Group on both Fp1 [*F*_(1, 16)_ = 6.08, *p* = 0.025] and Fp2 [*F*_(1, 16)_ = 8.50, *p* = 0.010], indicating that this activity was consistently larger in the fatigued cohort (5.47 μV ± 1.3 at Fp1 and 3.58 μV ± 1.5 at Fp2) than the non-fatigued cohort (2.37 μV ± 1.06 at Fp1 and −1.10 μV ± 1.1 at Fp2).

To summarize, the participants who reported a stronger increase in effort during the course of the task showed earlier and larger MRCP, along with long lasting prefrontal activity during the pre-motor phase.

#### Analysis based on MT

In the BP time window, a main effect of Block [*F*_(1, 16)_ = 9.53, *p* = 0.007] and a significant interaction [*F*_(1, 16)_ = 5.74, *p* = 0.029] was found, indicating that the fatigued cohort increased the BP amplitude from the early to late blocks, whereas the non-fatigued cohort did not show any differences across the fatiguing contractions. Similarly, analysis of the NS' showed a main effect of Block on Cz [*F*_(1, 16)_ = 6.43, *p* = 0.022], with a larger amplitude during the late rather than early blocks. The MRCP onset latency and the MP, and pP amplitudes did not differ between Group and Block.

#### Topographical maps

**Figure 4:**
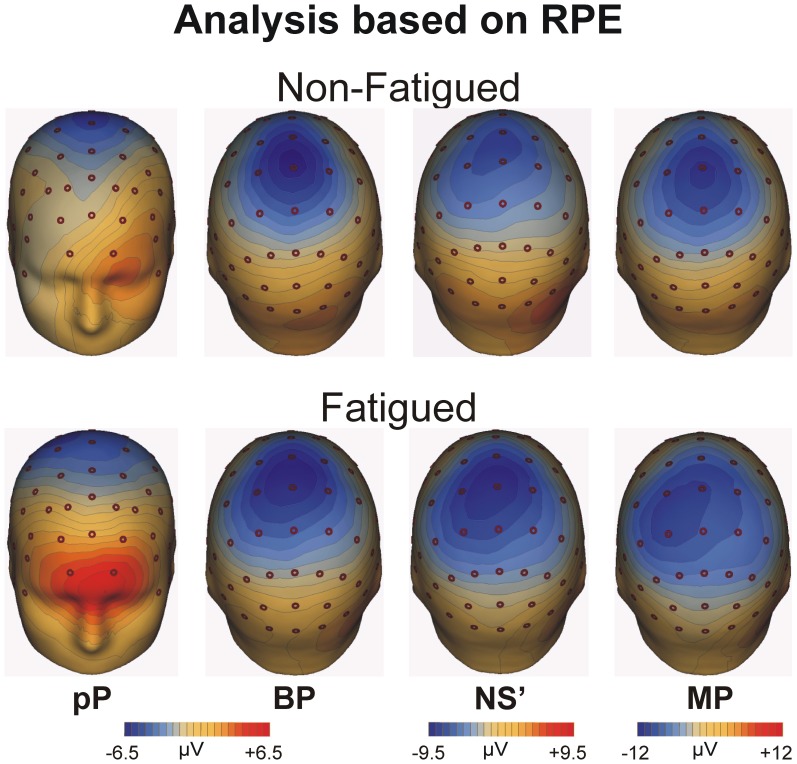
**Topographical maps of the MRCP averaged across all blocks for the fatigue and non-fatigued RPE cohorts for the pP, BP, NS', and MP peak activity**.

Topographical maps of the grand averaged MRCP components based on the rating of perceived effort for the non-fatigued (top) and fatigued (bottom) cohorts averaged across all blocks are shown in Figure 4. The maps are displayed from left to right for each representative component. The pP was particularly evident in the fatigued cohort and focused on the medial prefrontal cortex. The BP was distributed around the medial precentral areas, while the NS' and the MP were more posterior and contralateral to the used leg.

#### Correlation analysis

A significant positive correlation was found between the RPE and BP, RPE and NS', and RPE and pP amplitudes (*r* = 0.488, *p* = 0.044; *r* = 0.482, *p* = 0.048; *r* = 0.574, *p* = 0.013, respectively). The MP amplitude showed no significant correlations with the RPE. The MT did not show any correlation with the MRCP components.

## Discussion

We used a new experimental approach to assess the neurophysiological mechanisms underpinning fatigue during lower limb isometric contractions task for the first time. We confirmed that pre-motor brain activity increases with muscle fatigue (Johnston et al., 2001; Liu et al., 2005; Schillings et al., 2006; Falvo et al., 2011) and perceived effort (Slobounov et al., 2004; De Morree et al., 2012). The MRCP components were studied in the three distinct periods of motor preparation, initiation and execution, underpinning the perception of effort and muscle fatigue that was lacking in previous studies. Indeed, Liu et al. (2005) failed to observe any changes during the motor preparation process, Schillings et al. (2006) did not distinguish between the NS' and the MP, and De Morree et al. (2012) reported an increase in MRCP only during movement execution. The present findings show that muscle fatigue modulates activity in the supplementary and premotor areas as indexed by the larger amplitude of the BP and NS' in the fatigued MT cohort with respect to the non-fatigued MT cohort. On the other hand, the neural activity underpinning the perception of effort involves the supplementary and premotor areas, the primary motor cortex (MP) and, remarkably, the prefrontal areas (pP).

Comparison of the spatiotemporal MRCP pattern reported here for isometric contractions of lower limbs with previous literature on upper limbs (e.g., Slobounov et al., 2004; De Morree et al., 2012) makes it apparent that the NS' and MP components reported in the current study have a medial topographical distribution. This is likely due to a more medial representation of lower limbs in the motor cortex. Future studies employing the present paradigm on upper limbs may allow direct comparisons. However, the prefrontal activity has never been reported in previous MRCP studies addressing fatigue.

The large positive activity in the prefrontal cortex, which emerges during movement preparation, is only observed in participants reporting a high perception of effort and represents a novel result. The prefrontal cortex is not only involved in abstract planning, focused attention and executive/inhibitory functions (see Teffer and Semendeferi, 2012 for a review) but also in working memory processes. In the present study, the participants were asked to perform 240 isometric contractions with the right quadriceps at 40% of their MVC based on visual feedback of their force output provided by an oscilloscope. The isometric contraction of a large muscle group requires continuous adjustment of force output, which is associated with information processing between the brain and peripheral system, fine motor control, high executive/inhibitory processing during the planning of the contractions, visuo-motor integration and high levels of attention. Repetitive isometric contractions affect the steady-state of the internal environment, which results in the sensation of fatigue (Ament and Verkerke, 2009). Thus, peripheral fatigue occurring during this task is associated with central fatigue arising from the high cognitive processing that is required to correctly perform the task and emotional factors, such as the levels of motivation and attention. The prefrontal positivity observed in the fatigued RPE cohort could be the result of the high cognitive effort required to plan those actions.

It has been hypothesized (St. Clair Gibson et al., 2003) that the conscious perception of the sensation of fatigue arises from the prefrontal cortex, where current activities are compared with previous activities as a part of the decision-making processes to produce the required contraction intensity. Other areas are involved, such as the amygdala and the hippocampus, which have key roles in the emotional state, and the hypothalamus, brainstem and spinal cord, which are part of the metabolic regulatory system between afferent input and efferent commands. Furthermore, experimental data have indicated that schizophrenic patients have an abnormal awareness of effort caused by cerebral anomalies in the prefrontal areas (Lafargue and Franck, 2009), while data on healthy young adults reveal that the awareness of not being able to accomplish the action (i.e., impossible grasping) activates the prefrontal areas (Bozzacchi et al., 2012a).

The perception of effort has been defined as a sense of innervations (Proske, 2005) that are centrally generated by forwarding corollary discharges or afferent copies (Poulet and Hedwig, 2007) from the motor to sensory areas of the cerebral cortex by a corticofugal feedback system (Enoka and Stuart, 1992). Thus, it is difficult to localize the sensation of effort and fatigue within a single area of the brain. In addition, no regional classification of the brain is correct for any function (St. Clair Gibson et al., 2003). Based on present and previous findings, we can speculate that the development of the sensation of fatigue may lead to a short-term reorganization within the prefrontal-motor network during changes in the forward model and motor awareness (Desmurget and Sirigu, 2009). Furthermore, the awareness of the perception of effort may be built within the brain areas that are responsible for movement planning and control (i.e., frontal and prefrontal areas) prior to the action and regardless of muscle fatigue. In particular, the primary motor cortex controls voluntary muscle contractions and increases motor drive with fatigue, whereas the prefrontal cortex plays an important role in the creation of an awareness of the sensation of fatigue and the perception of effort during exercise. Taken together, the present findings could improve our knowledge regarding: (1) the temporal and topographical brain activities during lower limb action; (2) the neural activities underpinning both muscle fatigue and the perception of effort during a fatiguing task; and (3) the identification of the brain areas responsible for the awareness of the perception of effort. The perception of effort is the fundamental component of self-experience and is involved in the neuro-cognitive process of agency. Given the importance of the perception of effort in different research fields and pathologies, further studies are needed to address the neuro-anatomical structures and brain networks that generate the sense of effort, conscious sensation of fatigue, and origin of the corollary discharge by means of different recording techniques during ecological tasks.

### Conflict of interest statement

The authors declare that the research was conducted in the absence of any commercial or financial relationships that could be construed as a potential conflict of interest.

## References

[B1] Allen D. G. (2004). Skeletal muscle function: role of ionic changes in fatigue, damage and disease. Clin. Exp. Pharmacol. Physiol. 31, 485–493 10.1111/j.1440-1681.2004.04032.x 15298539

[B2] Allman B. L. Rice C. L. (2003). Perceived exertion is elevated in old age during an isometric fatigue task. Eur. J. Appl. Physiol. 89, 191–197 10.1007/s00421-002-0780-4 12665984

[B3] Ament W. Verkerke G. J. (2009). Exercise and fatigue. Sports Med. 39, 389–422 19402743 10.2165/00007256-200939050-00005

[B4] Barry B. K. Enoka R. M. (2007). The neurobiology of muscle fatigue: 15 years later. Integr. Comp. Biol. 47, 465–473 10.1093/icb/icm047 21672855

[B5] Berchicci M. Lucci G. Pesce C. Spinelli D. Di Russo F. (2012). Prefrontal hyperactivity in older people during motor planning. Neuroimage 62, 1750–1760 10.1016/j.neuroimage.2012.06.031 22732557

[B6] Borg G. A. V. (1982). Psychophysical bases of perceived exertion. Med. Sci. Sports Exerc. 14, 377–381 7154893

[B7] Borg G. A. V. (1990). Psychophysical scaling with applications in physical work and the perception of exertion. Scand. J. Work. Environ. Health 16, 55–58 10.5271/sjweh.1815 2345867

[B8] Borg G. A. V. (1998). Borg's Perceived Exertion and Pain Scales. Champaign, IL: Human Kinetics

[B9] Bozzacchi C. Giusti M. A. Pitzalis S. Spinelli D. Di Russo F. (2012a). Awareness affects motor planning for goal-oriented actions. Biol. Psychol. 89, 503–514 10.1016/j.biopsycho.2011.12.020 22234365

[B10] Bozzacchi C. Giusti M. A. Pitzalis S. Spinelli D. Di Russo F. (2012b). Similar cerebral motor plans for real and virtual actions. PLoS ONE 7:e47783 10.1371/journal.pone.0047783 23112847 PMC3480397

[B11] De Morree H. M. Klein C. Marcora S. M. (2012). Perception of effort reflects central motor command during movement execution. Psychophysiology 49, 1242–1253 10.1111/j.1469-8986.2012.01399.x 22725828

[B12] Desmurget M. Sirigu A. (2009). A parietal-premotor network for movement intention and motor awareness. Trends Cogn. Sci. 13, 411–419 10.1016/j.tics.2009.08.001 19748304

[B13] Dirnberger G. Duregger C. Trettler E. Lindinger G. Lang W. (2004). Fatigue in a simple repetitive motor task: a combined electrophysiological and neuropsychological study. Brain Res. 1028, 26–30 10.1016/j.brainres.2004.08.045 15518638

[B14] Enoka R. M. Duchateau J. (2008). Muscle fatigue: what, why and how it influences muscle function. J. Physiol. 586, 11–23 10.1113/jphysiol.2007.139477 17702815 PMC2375565

[B15] Enoka R. M. Stuart D. G. (1992). Neurobiology of muscle fatigue. J. Appl. Physiol. 72, 1631–1648 1601767 10.1152/jappl.1992.72.5.1631

[B16] Falvo M. J. Sirevaag E. J. Rohrbaugh J. W. Earhart G. M. (2011). Central adaptations to repetitive grasping in healthy aging. Brain Topogr. 24, 292–301 10.1007/s10548-011-0183-0 21519868 PMC3171517

[B17] Freude G. Ullsperger P. (1987). Changes in Bereitschaftspotential during fatiguing and non-fatiguing hand movements. Eur. J. Appl. Physiol. Occup. Physiol. 56, 105–108 3830133 10.1007/BF00696384

[B18] Gandevia S. C. (2001). Spinal and supraspinal factors in human muscle fatigue. Physiol. Rev. 81, 1725–1789 11581501 10.1152/physrev.2001.81.4.1725

[B19] Gratton G. Coles M. G. H. Donchin E. (1983). A new method for off-line removal of ocular artifact. Electroencephalogr. Clin. Neurophysiol. 55, 468–484 6187540 10.1016/0013-4694(83)90135-9

[B20] Haggard P. (2009). Neuroscience. The sources of human volition. Science 324, 731–733 10.1126/science.1173827 19423807

[B21] Heuser M. Pincivero D. (2010). The effects of stretching on knee flexor fatigue and perceived exertion. J. Sports Sci. 28, 219–226 10.1080/02640410903460718 20391093

[B22] Hotta Y. Ito K. (2011). EMG-based detection of muscle fatigue during low-level isometric contraction: effects of electrode configuration and blood flow restriction. Conf. Proc. IEEE Eng. Med. Biol. Soc. 2011, 3877–3879 10.1109/IEMBS.2011.6090963 22255186

[B23] Hummel A. Läubli T. Pozzo M. Schenk P. Spillmann S. Klipstein A. (2005). Relationship between perceived exertion and mean power frequency of the EMG signal from the upper trapezius muscle during isometric shoulder elevation. Eur. J. Appl. Physiol. 95, 321–326 10.1007/s00421-005-0014-7 16096843

[B24] Johnston J. Rearick M. Slobounov S. (2001). Movement-related cortical potentials associated with progressive muscle fatigue in a grasping task. Clin. Neurophysiol. 112, 68–77 11137663 10.1016/s1388-2457(00)00452-1

[B25] Kristeva R. (1977). Study of the motor potential during voluntary recurrent movement. Electroencephalogr. Clin. Neurophysiol. 42, 588 10.1016/j.ejpain.2010.01.001 20181504

[B26] Lafargue G. Franck N. (2009). Effort awareness and sense of volition in schizophrenia. Conscious. Cogn. 18, 277–289 10.1016/j.concog.2008.05.004 18653358

[B27] Lambert E. V. St. Clair Gibson A. Noakes T. D. (2005). Complex systems model of fatigue: integrative homoeostatic control of peripheral physiological systems during exercise in humans. Br. J. Sports Med. 39, 52–62 10.1136/bjsm.2003.011247 15618343 PMC1725023

[B28] Liu J. Z. Yao B. Siemionow V. Sahgal V. Wang X. Sun J. (2005). Fatigue induces greater brain signal reduction during sustained than preparation phase of maximal voluntary contraction. Brain Res. 1057, 113–126 10.1016/j.brainres.2005.07.064 16129419

[B29] Macaluso A. De Vito G. Felici F. Nimmo M. A. (2000). Electromyogram changes during sustained contraction after resistance training in women in their 3rd and 8th decades. Eur. J. Appl. Physiol. 82, 418–424 10.1007/s004210000212 10985596

[B30] Menotti F. Bazzucchi I. Felici F. Damiani A. Gori M. C. Macaluso A. (2012). Neuromuscular function after muscle fatigue in Charcot-Marie-Tooth type 1A patients. Muscle Nerve 46, 434–439 10.1002/mus.23366 22907236

[B31] Pincivero D. M. (2011). Older adults underestimate RPE and knee extensor torque as compared with young adults. Med. Sci. Sports Exerc. 43, 171–180 10.1249/MSS.0b013e3181e91e0d 20508539

[B32] Popivanov D. Mineva A. Dushanova J. (1995). Single-trial readiness potentials and fatigue. Adv. Exp. Med. Biol. 384, 295–304 8585459 10.1007/978-1-4899-1016-5_23

[B33] Poulet J. F. Hedwig B. (2007). New insights into corollary discharges mediated by identified neural pathways. Trends Neurosci. 30, 14–21 10.1016/j.tins.2006.11.005 17137642

[B34] Preston J. Wegner D. M. (2009). Elbow grease: when action feels like work, in Oxford Handbook of Human Action, eds Morsella E. Bargh J. A. Gollwitzer P. M. (Oxford, UK: Oxford University Press), 569–586

[B35] Proske U. (2005). What is the role of muscle receptors in proprioception? Muscle Nerve 31, 780–787 10.1002/mus.20330 15818635

[B36] Schillings M. L. Kalkman J. S. van der Werf S. P. Bleijenberg G. van Engelen B. G. Zwarts M. J. (2006). Central adaptations during repetitive contractions assessed by the readiness potential. Eur. J. Appl. Physiol. 97, 521–526 10.1007/s00421-006-0211-z 16718505

[B37] Shibasaki H. Hallett M. (2006). What is Bereitschaftspotential? Clin. Neurophysiol. 117, 2341–2356 10.1016/j.clinph.2006.04.025 16876476

[B38] Siemionow V. Fang Y. Calabrese L. Sahgal V. Yue G. H. (2004). Altered central nervous system signal during motor performance in chronic fatigue syndrome. Clin. Neurophysiol. 115, 2372–2381 10.1016/j.clinph.2004.05.012 15351380

[B39] Sjøgaard G. Kiens B. Jørgensen K. Saltin B. (1991). Intramuscular pressure, EMG and blood flow during low-level prolonged static contraction in man. Brain 114, 85–98 3788624 10.1111/j.1748-1716.1986.tb08002.x

[B40] Slobounov S. Hallett M. Newell K. M. (2004). Perceived effort in force production as reflected in motor-related cortical potentials. Clin. Neurophysiol. 115, 2391–2402 10.1016/j.clinph.2004.05.021 15351382

[B41] Søgaard K. Gandevia S. C. Todd G. Petersen N. T. Taylor J. L. (2006). The effect of sustained low-intensity contractions on supraspinal fatigue in human elbow flexor muscles. J. Physiol. 573, 511–523 10.1113/jphysiol.2005.103598 16556656 PMC1779725

[B42] St. Clair Gibson A. Baden D. A. Lambert M. I. Lambert E. V. Harley Y. X. R. Hampson D. (2003). The conscious perception of the sensation of fatigue. Sports Med. 33, 167–176 12656638 10.2165/00007256-200333030-00001

[B43] Suetta C. Aagaard P. Rosted A. Jakobsen A. K. Duus B. Kjaer M. (2004). Training-induced changes in muscle CSA, muscle strength, EMG, and rate of force development in elderly subjects after long-term unilateral disuse. J. Appl. Physiol. 97, 1954–1961 10.1152/japplphysiol.01307.2003 15247162

[B44] Taylor J. L. Gandevia S. C. (2008). A comparison of central aspectsof fatigue in submaximal and maximal voluntary contractions. J. Appl. Physiol. 104, 542–550 10.1152/japplphysiol.01053.2007 18032577

[B45] Teffer K. Semendeferi K. (2012). Human prefrontal cortex: evolution, development, and pathology. Prog. Brain Res. 195, 191–218 10.1016/B978-0-444-53860-4.00009-X 22230628

[B46] Zijdewind I. Zwarts M. J. Kernell D. (1998). Influence of a voluntary fatigue test on the contralateral homologous muscle in humans? Neurosci. Lett. 253, 41–44 10.1016/S0304-3940(98)00609-0 9754800

[B47] Zwarts M. J. Bleijenberg G. van Englen B. G. M. (2008). Clinical neurophysiology of fatigue. Clin. Neurophysiol. 119, 2–10 10.1016/j.clinph.2007.09.126 18039594

